# Long-Term Outcome of Surgery for Perianal Crohn’s Fistula

**DOI:** 10.3390/medicina60071035

**Published:** 2024-06-24

**Authors:** Marie Schaad, Alain Schoepfer, Jean-Benoît Rossel, Mamadou Pathé Barry, Gerhard Rogler, Dieter Hahnloser

**Affiliations:** 1 Service of Visceral Surgery, Lausanne University Hospital (CHUV), University of Lausanne, Bugnon 46, 1005 Lausanne, Switzerland; 2 Service of Gastroenterology, Lausanne University Hospital (CHUV), University of Lausanne, Bugnon 46, 1005 Lausanne, Switzerland; 3 Unisanté, University Center for Primary Care and Public Health, University of Lausanne, 1011 Lausanne, Switzerland; 4 Service of Gastroenterology and Hepatology, University Hospital Zürich, University of Zürich, 8091 Zurich, Switzerland

**Keywords:** perianal fistula, Crohn’s disease, Seton drainage

## Abstract

*Background and Objectives*: Patients with perianal Crohn’s (CD) fistula often need repetitive surgeries and none of the established techniques was shown to be superior or preferable. Furthermore, the long-term outcome of fistula Seton drainage is not well described. The aims of this study were to analyze the long-term healing and recurrence rate of CD perianal fistulas in a large patient cohort. *Materials and Methods*: Database analysis of the Swiss IBD (Inflammatory Bowel Disease) cohort study. *Results*: 365 perianal fistula patients with 576 surgical interventions and a median follow-up of 7.5 years (0–12.6) were analyzed. 39.7% of patients required more than one procedure. The first surgical interventions were fistulectomies ± mucosal sliding flap (59.2%), Seton drainage (29.6%), fistula plugs or fibrin glue installations (2.5%) and combined procedures (8.8%). Fistulectomy patients required no more surgery in 69%, one additional surgery in 25% and more than one additional surgery in 6%, with closure rates at 7.5 years follow-up of 77.1%, 74.1% and 66.7%, respectively. In patients with Seton drainage as index surgery, 52% required no more surgery, and over 75% achieved fistula closure after 10 years. *Conclusions*: First-line fistulectomies, when feasible, achieved the highest healing rates, but one-third of patients required additional surgeries, and one-fourth of patients will remain with a fistula at 10 years. Initial Seton drainage and concurrent medical therapy can achieve fistula closure in 75%. However, in 50% of patients, more surgeries are needed, and fistula closure is achieved in only two-thirds of patients.

## 1. Introduction

Crohn’s disease (CD) is an inflammatory disease of the gastrointestinal tract, that affects the integrity of the intestinal wall through transmural inflammation. This condition can result in serious complications like perianal abscesses and fistulas [1].

Perianal fistulas comprise a frequent and debilitating complication of CD. Indeed, overall, 30–50% of patients with CD are affected [2,3,4], being the initial manifestation of CD in 10% of patients [3]. Its cumulative incidence can reach 22% at 10 years and 26% after 20 years [5]. The symptoms caused by CD perianal fistulas may include anal pain, swelling, fever, fatigue, bowel urgency and fecal incontinence [1,6,7]. Therefore, it is a challenging condition to manage, often leading to higher rates of surgery when compared to individuals with Crohn’s disease not affecting the perianal area. Additionally, it significantly impacts quality of life, personal well-being, relationships and professional opportunities [1,7,8].

Clinical evaluation, imaging, and examination under anaesthesia (EUA) are essential for diagnosis. EUA by an experienced surgeon is 90% accurate for detecting and classifying perianal fistulas and abscesses [9,10]. Pelvic MRI remains the gold standard in perianal CD [9,11]. Alternatives include endoanal ultrasound, which is useful but less accurate than MRI [10], and transperineal ultrasound, a non-invasive, cost-effective option with comparable accuracy to MRI. Combining two imaging modalities enhances accuracy to nearly 100% [8,12]. Indeed, a proper diagnostic approach is crucial to avoid incomplete healing or sphincter damage [13].

During the course of the disease, multiple medical treatments are used but often surgery is required: up to 90% of patients will need a surgical intervention at least once [14,15]. To optimize the treatment plan and achieve fistula healing, a multidisciplinary approach is necessary [16,17]. The first line of surgical treatment frequently is a Seton drainage [18], which is a drain inserted through the fistula that keeps fistulous tracts open for drainage. It prevents sepsis, promotes fibrosis and preserves the external anal sphincter, reducing recurrent abscesses [9,19]. In combination with anti-TNF agents, Seton drainage can sometimes be the definitive treatment but a consensus on the optimal duration of insertion of the Seton is lacking [4,17]. After removal, there is a recurrence of the fistula in 40–80% of patients [20]. Currently, none of the surgical techniques has shown to be superior in terms of healing rates. 

Reparative surgical options can be grouped into minor vs. major. Minor options include a fistula plug [9,21], a device made of collagen or porcine intestinal submucosa that fills the fistula tract closing the internal opening, and fibrin glue instillation [20], the curettage of the fistula canal followed by closure with synthetic glue or fibrin. The other surgical options are considered major and include fistulectomy/fistulotomy, the opening of the fistula canal along its length taking careful consideration of the sphincter anatomy [9], and mucosal sliding flap, a flap mobilised from the rectum and used to close the internal opening of the fistulous track, thus allowing the external opening to drain and heal [13,22]. 

The choice of procedure considers the anatomical structure of the fistula, as well as the surgeon’s skills and experience. Among patients requiring surgery, the greatest risk is damage to the sphincter, leading to incontinence, and recurrence, with the need for reoperation [23,24].

The aims of this study were to analyse the long-term healing and recurrence rates of perianal fistulas in CD patients, stratified according to the first procedure performed and to determine the outcome of Seton drainage. 

## 2. Materials and Methods

This is a retrospective analysis of the prospective Swiss Inflammatory Bowel Disease Cohort Study (SIBDCS) [25]. The study protocol was approved by the scientific committee of the SIBDCS on 2 April 2019 (SIBDCS Project N°2019-08). The SIBDCS has collected data on Inflammatory Bowel Disease (IBD) patients in Switzerland since 2006, and the aim is to improve the understanding of CD [25,26]. At a first assessment, data are collected retrospectively, then updated yearly via patient and physician-based questionnaires and/or examination. 

The study included all adult patients with Crohn’s disease who have a perianal fistula (n = 365) and who participated in the SIBDCS. There were no exclusion criteria. The primary outcome of the study was the healing rate of perianal fistula defined as no external opening present on clinical examination and no clinical complaints of anal pain, swelling and/or oozing. Secondary outcomes were the need and type for re-surgery and the re-insertion and /or presence of a Seton drainage on long-term follow-up. The following parameters of the cohort were analysed: patient demographics (gender, age, smoking status), Crohn’s related data (location of disease, current severity of Crohn’s disease (CDAI), mucosal healing at last colonoscopy, past and current medical therapies) and surgical procedures (categorized as fistula plug/fibrin glue instillation, fistulectomy/fistulotomy/mucosal sliding flap and Seton drainage). Follow-ups of fistula were categorized as surgical therapy, steady state, new/reopening, improvement (all defined as unhealed) and closure (defined as healed).

Patients were grouped according to the type of first surgery into four groups: Seton drainage only (no fistula reconstructive surgery), Fistulectomy ± Mucosal sliding flap (reconstructive surgery), Seton drainage + Fistulectomy/Mucosal sliding flap (combination of the first and second group for patients with multiple fistula) and Fistula plug or fibrin glue instillation.

### Statistical Analysis

Clinical data were retrieved at the SIBDCS data center. All standard statistical analyses were performed using STATA^Ò^ (Version 16.0, College Station, TX, USA). Continuous data distribution was analysed using normal QQ-plots. For continuous data, results are presented as median and range in the case of non-Gaussian data and as mean ± SD and range in the case of Gaussian data. For categorical data, results are presented as absolute numbers and relative frequencies. 

The Kaplan-Meier method was used to examine cumulative occurrence of fistula and surgery over time; Turnbull’s extension to the Kaplan–Meier method was used for left- and interval-censored data [27]. This particular methodology allows the assessment of the frequency of events at distinct time points in a cohort that is not population-based, but that is referral-based.

## 3. Results

The study included 365 patients (54% male, median age of 32 years) with 576 interventions and a median follow-up of 7.5 years (range 0–12.6). 27.1% of patients had only rectal CD. 

The type of first surgery and patients’ demographics are listed in Table 1. 

Two hundred and twenty (60.3%) patients had only one surgery, and 39.7% of patients required at least one additional surgery. One hundred and seven (29.3%) patients needed a second surgery and 38 (10.45%) patients required two or more additional surgeries. The median time to the second surgery was 2.0 years, 4.8 years until the third surgery and 5.1 years until the fourth. 

Fistulectomy ± mucosal sliding flap as first intervention had the highest overall healing rate of 75%; however, 31% of the patients with fistulectomy required a second or third surgery (Table 2).

Seton drainage as first intervention had a 60% healing rate but required in 48% of cases a second intervention, whereas a fistula plug or fibrin glue instillation as first intervention healed fistulas only in 55%.

At the last follow-up, 255 (69.9%) patients had a healed fistula (Table 2). There was no significant difference in patients ever receiving anti-TNF therapy (94.7% healed vs. 98.5% unhealed, *p* = 0.1), nor currently being under anti-TNF therapy (45.6% vs. 41.9%, *p* = 0.619), nor presenting with clinical remission (=CDAI < 150; 96.5% vs. 98.5%; *p* = 0.339). Not surprisingly, mucosal healing at last colonoscopy was significantly higher in the healed fistula group (70.2% vs. 28.8%, *p* = 0.001).

A total of 165 of 365 patients (45.2%) had a Seton at any time, regardless of alone or in combination with another procedure and regardless of it being the first (140 (84.8%) patients) or following procedure (25 (15.2%) patients). The median age at the time of the Seton procedure was 31.7 years (13.4–74.0). A total of 58.8% of patients were male and 25.7% were smokers, and 33.9% of patients had only perianal CD. There was no significant difference in patient demographics with a healed or unhealed fistula at the last follow-up (Table 3), except for the CDAI score, which was significantly higher in patients with an unhealed fistula (6 (0–182) vs. 33 (0–217), *p* = 0.0001).

**Figure 1:**
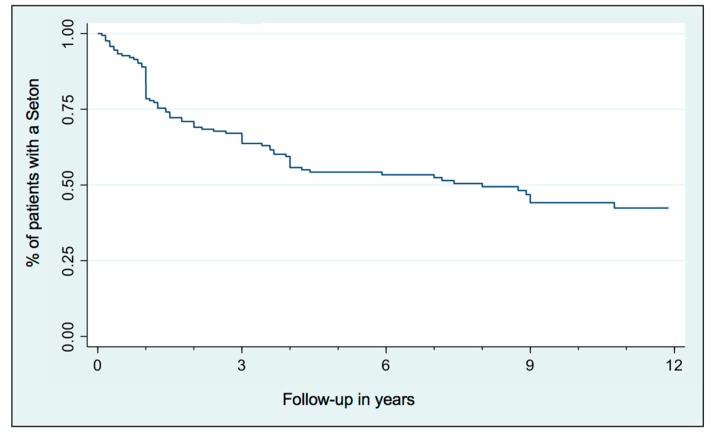
Follow-up (years) of Seton-free survival.

**Figure 2:**
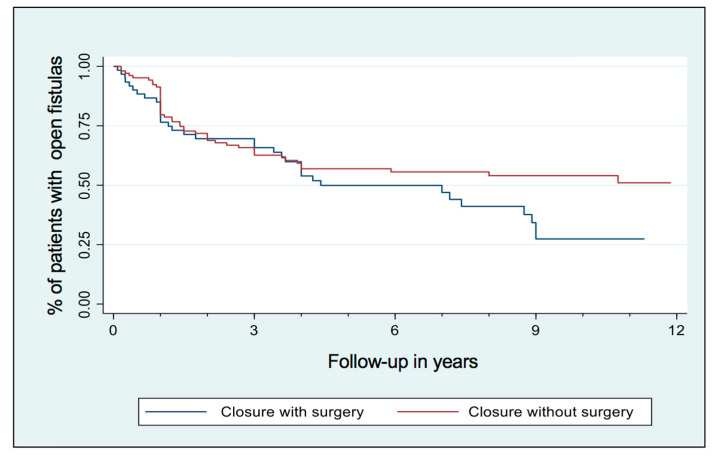
Fistula healing depending on surgery, for all patients with a Seton.

**Figure 3:**
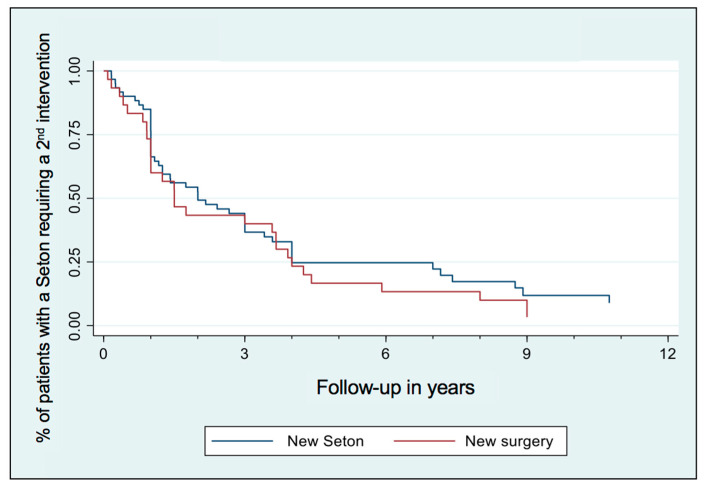
Patients with Seton requiring another intervention: timing until either new surgery or new Seton.

Nearly 50% of patients having a Seton remain with it over time (Figure 1). 49.7% of patients with a Seton required another surgery after it (fistula repair surgery or new Seton). Seton removal can heal the fistula in approximatively 50% of patients over time, whereas additional surgery increased the closure rate by another 25% (Figure 2). Nevertheless, healing takes time and the majority of patients required additional Setons and/or surgeries (Figure 3). 

## 4. Discussion

In this retrospective analysis of the prospective SIBDCS, first-line fistulectomy/mucosal sliding flap, if feasible, achieved the highest healing rates in perianal CD. However, one-third of patients required other additional surgeries, and approximately one in four patients will remain with a fistula at 10 years. Moreover, with Seton drainage as first-line surgery and concurrent medical therapy, fistula closure can be achieved in 50% of patients, without requiring further intervention. 

A recent Danish study, which included 1812 patients with perianal CD, estimated that 84% of them required surgery, with a median of 2 procedures [15]. Our study also found that 39.7% of the patients require multiple surgeries, in accordance with published literature, which found that a large proportion of patients need several operations in order to increase the healing rate [28,29].

As stated by the 3rd European Evidence-based Consensus on the Diagnosis and Management of Crohn’s Disease, an essential point is also that “active luminal CD should be treated if present, in conjunction with appropriate surgical management of fistulae” [23]. We found a concordant result with a mucosal healing rate of 70.2% in the healed fistula group versus 28.8% in the unhealed group (*p* = 0.001), this at last colonoscopy, which demonstrates the need for control of mucosal inflammation prior to intervention. 

Regarding the various surgical treatment techniques, we found that fistulectomy/mucosal sliding flap achieved a primary healing rate of 69%, which is the highest among the different options included in our study. This finding is comparable to a literature review performed between 1978 and 2008, that identified 1654 patients who underwent an endorectal advancement flap, with a success rate of 64% [22]. Depending on the anatomy of the fistula, the surgeon’s knowledge and the patient’s choice, this option should, therefore, be tried first.

On the contrary, minor surgery options, such as fistula plugs or fibrin glue instillations, have not shown good results, despite a very low number of patients, which would have to be confirmed by larger cohorts. In a French multi-centre, open-label, randomized controlled trial comparing Seton removal alone with anal fistula plug insertion on 106 CD patients, the fistula plug was not more effective than Seton removal alone to achieve fistula closure (31.5% vs. 23.1%, *p* = 0.19) [30]. 

Concerning Seton drainages, the PISA randomised trial compared chronic Seton drainage to anti-TNF treatment for one year and to surgical closure with a two-month anti-TNF, in patients with CD perianal fistula with a single internal opening [31]. They concluded that Seton treatment alone was associated with a higher re-intervention rate (10/15 vs. 6/15 anti-TNF vs. 3/14 surgical closure, *p* = 0.02), without any noticeable difference in perianal disease activity, nor quality of life between the three groups. 

In our study, among the 165 patients who had a Seton, 50% of them kept an indwelling Seton chronically for several months. 50% of patients present mucosa healing after only removal of the Seton, which also demonstrates the essential importance of the combination with medical treatment. We found better results concerning patients with Seton drainage alone because of a probable positive selection bias with well-selected patients. For patients requiring a second intervention, it happens on average 1.4 years after the first one and allows, in the long term, to increase the healing rate by 25%. 

For the first 5 years of follow-up, we noticed that the two curves “closure with vs. without surgery” were quite parallel: the follow-up depends on the choice of treatment for and by the patient; unfortunately, surgical indications are not included in our database. However, an important point, also found in the literature [3,17,31], is the importance of multidisciplinary decision-making, including the patient and the various specialists.

This study also has limitations, which were mainly the inequalities of numbers between the different types of interventions despite a very large number of patients. Due to the retrospective collection of register data, we were limited to the information contained in the database, which did not allow us to consider the anatomical location of fistulas and/or Parks’ classification. Moreover, specific data regarding the indication for the initial intervention and/or re-operation were not available. Nevertheless, the main strength of this study is its long-term follow-up with at least one visit per year and a cohort of patients from all over Switzerland representing a national standard of care. Therefore, these data are essential as they can be generalized to the population and patients can be counselled using these figures. 

## 5. Conclusions

This study shows that first-line fistulectomy/mucosal sliding flap achieved the highest healing rates in perianal CD, hence should be tried first, if allowed by the fistula anatomy and the surgeon’s skills. However, one-third of patients required other additional surgeries and approximately one in four patients will remain with a fistula at 10 years. Initial Seton drainage and concurrent medical therapy can achieve fistula closure in 75%, demonstrating the importance of controlling mucosal inflammation. 

## Figures and Tables

**Table 1:** Patient demographics according to type of first perianal fistula surgery.

First Surgery	n	Male	Median Age (Range) at First Surgery	Only Perineal Location of Crohn	Perineal and Other Location
Seton drainage only	108 (29.6%)	63 (58.3%)	31.5 (13.4–74.0)	36 (33.3%)	72 (66.7%)
Fistulectomy ± Mucosal sliding flap	216 (59.2%)	108 (50%)	31.9 (4–80)	52 (24.1%)	162 (75%)
Seton drainage + Fistulectomy/Mucosal sliding flap	32 (8.8%)	20 (62.5%)	30.9 (14–51.7)	10 (31.25%)	22 (68.75%)
Fistula plug or fibrin glue instillation	9 (2.5%)	6 (66.7%)	50.4 (19.3–64)	1 (11.1%)	8 (88.9%)
Total	365 (100%)	197 (54%)	31.9 (4–80)	99 (27.1%)	264 (72.3%)

**Table 2:** Healed fistulas at last follow-up according to the type of first surgery and the need of additional surgeries.

First Surgery	Patients with a Healed Fistula at Last Available Follow-Up	Median Follow-Up (Range), in Years	No Additional Surgery	One Additional Surgery		2 or More Additional Surgery	
			n	n	Median Time to (yrs, Range)	n	Median Time to (yrs, Range)
Seton drainage	65/108 (60%)	8.3 (1–27)	34 (52%)	23 (35%)	5.9 (2.5–18)	8 (12%)	7.8 (4–10)
Fistulectomy ± Mucosal sliding flap	162/216 (75%)	7.4 (0.5–4)	111 (69%)	41 (25%)	13.8 (0.8–42)	10 (6%)	13.3 (6–26)
Seton drainage + Fistulectomy/ Mucosal sliding flap	23/32 (72%)	7.3 (2–18)	15 (65%)	6 (26%)	6.9 (3–10)	2 (9%)	7.3 (4–11)
Fistula plug or fibrin glue instillation	5/9 (55%)	2.8 (2–3)	4 (80%)	1 (20%)	6	0 (0%)	-

**Table 3:** Patient demographics at last follow-up with Seton drainage.

	Healed Fistula (n = 104)	Unhealed Fistula (n = 61)	*p*-Value
Median follow-up (range), in years	8.1 (0.4–27.2)	5.8 (0.2–34.2)	0.10
Age at Seton drainage (median, range)	31.8 (13.4–74.0)	31.7 (14.7–58.7)	0.56
Male gender	62 (59.6%)	35 (57.4%)	0.78
Only perineal location of Crohn	36 (34.6%)	20 (32.8%)	0.81
Percentage smoker	22/93 (23.7%)	17/59 (28.8%)	0.48
Current Crohn Disease Activity Index (median, range)	6 (0–182)	33 (0–217)	0.0001
Current Anti-TNF medication	50 (48.1%)	35 (57.4%)	0.25
Additional surgery (any)	46 (44.2%)	36 (59.0%)	0.45

## Data Availability

The SIBDC data can be accessed by all investigators of the SIBDCS. External investigators can submit research proposals to the scientific committee of the SIBDCS which will evaluate the quality of the project and allow access to selected data sets after approval and a signed data transfer agreement.
